# Digital Humanities in Child and Adolescent Mental Health Services: A Review

**DOI:** 10.3390/children13070967

**Published:** 2026-07-22

**Authors:** Saahoon Hong, Betty Walton, Hea-Won Kim

**Affiliations:** School of Social Work, Indiana University, Indianapolis, IN 46202, USAheakim@iu.edu (H.-W.K.)

**Keywords:** artificial intelligence, child and adolescent mental health, digital mental health interventions, ethics of care, humanities-informed approaches

## Abstract

**Highlights:**

**What are the main findings?**

- This scoping review identified six recurring domains through which digital humanities informs AI-enabled youth mental health interventions: engagement, participatory co-design, human oversight, equity, ethical governance, and implementation.
- Across the reviewed evidence, humanities-informed approaches consistently complemented AI development by incorporating relational, cultural, and ethical perspectives into digital mental health systems.

**What are the implications of the main findings?**

- Digital humanities offers an interdisciplinary framework that can guide the responsible design, governance, and implementation of AI-enabled mental health technologies for children and adolescents.
- Future research should empirically evaluate humanities-informed AI approaches, particularly in relation to implementation, equity, and emerging generative AI applications, to support ethical and developmentally responsive mental health systems.

**Abstract:**

Background/Objectives: Artificial intelligence (AI) is increasingly used in youth mental health services, including clinical decision support, risk prediction, and digital therapeutics. However, existing frameworks provide limited guidance for integrating ethical, cultural, and relational considerations into the design, governance, and implementation of AI-enabled mental health technologies. This scoping review examined how digital humanities-informed approaches have been incorporated into AI-supported mental health interventions for children and adolescents. Methods: A scoping review was conducted following the PRISMA Extension for Scoping Reviews (PRISMA-ScR) guidelines. Peer-reviewed literature published between 2015 and 2025 was searched using PubMed and supplemented by semantic searches through Elicit. Systematic reviews, scoping reviews, and meta-analyses examining AI-enabled digital mental health interventions and digital humanities perspectives were included. Data were synthesized using inductive thematic analysis. Results: Seventeen review-level studies met the inclusion criteria. Six recurring themes were identified: engagement, participatory co-design, human oversight, equity, ethical governance, and implementation. Across the included reviews, humanities-informed approaches were associated with greater attention to relational engagement, stakeholder participation, transparency, contextual adaptation, and culturally responsive implementation. Evidence supporting intervention effectiveness was strongest in systematic reviews and meta-analyses, whereas findings related to ethics, governance, equity, and implementation were derived primarily from scoping reviews and conceptual syntheses. Conclusions: This review suggests that digital humanities provides a valuable interdisciplinary perspective for informing the design, governance, and implementation of AI-enabled youth mental health interventions. Although the current evidence base remains heterogeneous, integrating humanities-informed approaches may support the development of AI systems that are more ethical, equitable, and developmentally responsive. Future research should evaluate these approaches through empirical implementation studies and emerging generative AI applications.

## 1. Introduction

Mental health services for children and adolescents face persistent structural strain. Rising prevalence of anxiety, depression, trauma exposure, and neurodevelopmental conditions has converged with workforce shortages, geographic inequities, and prolonged waitlists, producing substantial disparities in timely access to care [1,2,3]. In response, AI-enabled screening tools, telehealth platforms, mobile applications, and chatbot-based supports are increasingly deployed to mitigate these systemic barriers [4,5]. Meta-analytic evidence suggests that youth-focused digital mental health interventions (DMHIs) can yield statistically significant symptom reductions, particularly for social anxiety and depressive disorders, although effect sizes are often modest and moderated by guidance intensity, developmental stage, and engagement quality [6,7,8].

This growth, however, has outpaced its ethical and relational foundations. AI-supported technologies introduce risks related to privacy, algorithmic bias, opaque decision-making, and inequitable access, compounded by sociodemographic underreporting that limits equity analysis and external validity [9,10,11]. More fundamentally, automated agents lack the relational accountability and contextual discernment central to therapeutic care, a limitation with particular consequence for young people, whose mental health is not reducible to discrete symptom clusters but is lived within family systems, peer networks, cultural communities, and developmental trajectories [12,13,14]. When technological interventions abstract youth into data points, they risk obscuring precisely the social and narrative dimensions that developmental and ecological models identify as central to recovery [12,13,14]. These gaps point to the need for frameworks capable of foregrounding relational integrity, cultural context, and youth agency alongside technical performance [9], a need this review addresses through the lens of digital humanities.

Digital humanities is an interdisciplinary field integrating humanities theories and methods with computational and digital technologies to examine how technology shapes, and is shaped by, human culture, identity, ethics, communication, and power [15,16,17,18]. Within this framework, narrative medicine, digital storytelling, philosophy, cultural studies, and participatory design function not as independent domains but as complementary traditions offering interpretive, ethical, and sociocultural grounding for the design, evaluation, and governance of AI-enabled mental health systems [19,20,21,22]. This orientation converges with empirical findings already established in the digital mental health literature: systematic and scoping reviews consistently identify engagement, co-design, caregiver involvement, and cultural responsiveness as key determinants of intervention effectiveness [8,23,24]. Youth-centered co-design improves usability and acceptability, caregiver participation enhances retention and continuity of care, and developmental stage and relational supports moderate outcomes [7,23,24]—while persistent deficits in demographic reporting and cultural tailoring underscore the need for equity-informed design [11]. Read together, this evidence suggests that narrative, relational, and cultural factors are not peripheral to digital effectiveness but foundational to it, and that humanities traditions in narrative ethics, philosophy of psychiatry, and values-based practice offer the normative grounding—centered on dignity, autonomy, and interpretive meaning—needed to translate that insight into design and governance practice [14,25,26]. In AI-supported behavioral health, these perspectives help safeguard against reductive algorithmic approaches while preserving youth agency, particularly where children’s voices are mediated by caregivers, institutions, and data systems. Embedding narrative-informed approaches within digital platforms may further humanize care, reduce stigma, and prevent the reduction of young people to predictive risk profiles [27,28].Emerging applications illustrate this potential concretely: culturally co-designed tools developed with Asian American young adults show improved alignment with community narratives and values [29], and digital storytelling programs adapted to Indigenous healing traditions report enhanced engagement and culturally resonant outcomes [30]. Yet despite this promise, the evidence base remains fragmented—largely qualitative, small-scale, and embedded within broader digital programs, with barriers such as digital literacy disparities, uneven broadband access, workflow integration challenges, and concerns about triggering content still requiring systematic synthesis [31]. No comprehensive review has yet mapped how digital humanities methodologies are being operationalized within child and youth mental health systems, despite growing recognition that engagement and cultural context determine effectiveness.

The present review addresses this gap by mapping the existing literature on digital humanities-informed interventions within mental health service settings for children and adolescents. Specifically, it aims to (1) identify the digital humanities methods employed (e.g., digital storytelling, participatory media, arts-based technologies, AI systems incorporating narrative elements); (2) characterize the populations and service contexts in which these approaches are implemented; (3) synthesize reported outcomes, including impacts on stigma, engagement, empowerment, clinical indicators, and recovery processes; and (4) analyze how these interventions address ethical and cultural considerations and align with recovery-oriented principles. In doing so, the review critically interrogates themes with direct implications for AI governance in public-sector behavioral health systems—cultural responsiveness, youth agency, narrative legitimacy, and the ethics of algorithmic mediation—to inform policy, funding, and infrastructure planning that ensures technological transformation strengthens, rather than erodes, the relational and humanistic foundations of youth behavioral health care.

## 2. Methods

### Design

To identify relevant literature at the intersection of the humanities and AI-based digital mental health tools, this study searched PubMed for secondary research between January and February 2025. Search strings were applied to titles and abstracts, combining terms related to digital technologies, youth populations, mental health, and humanities. The following search strategy was used:

((“digital”[Title/Abstract] OR “artificial intelligence”[Title/Abstract] OR AI[Title/Abstract] OR “machine learning”[Title/Abstract] OR chatbot*[Title/Abstract] OR “large language model*”[Title/Abstract])

AND

(child*[Title/Abstract] OR youth[Title/Abstract] OR adolescent*[Title/Abstract])

AND

(“mental health”[Title/Abstract] OR depression[Title/Abstract] OR anxiety[Title/Abstract] OR wellbeing[Title/Abstract])

AND

(humanities[Title/Abstract] OR ethics[Title/Abstract] OR narrative[Title/Abstract] OR storytelling[Title/Abstract] OR philosophy[Title/Abstract] OR culture[Title/Abstract]))

**Figure 1:**
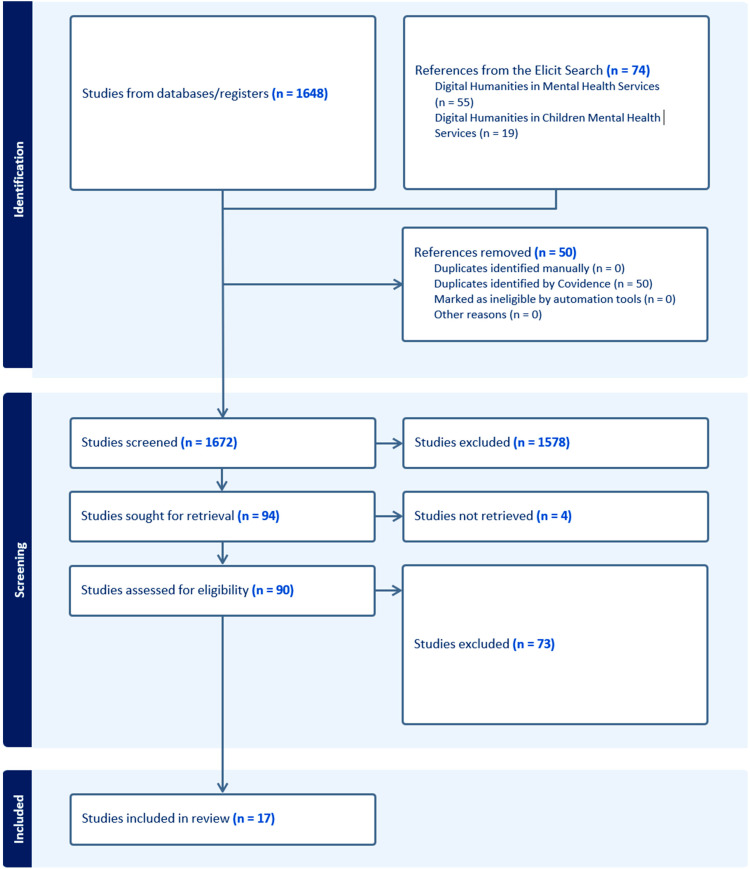
PRISMA flow diagram.

This search yielded 1648 records (Figure 1). To ensure interdisciplinary breadth, a supplementary semantic search was conducted using the Elicit engine [32], which draws from the Semantic Scholar and OpenAlex databases. The queries “Digital Humanities in Mental Health Services” and “Digital Humanities in Children Mental Health Services” were used to retrieve studies that explicitly address the integration of humanities disciplines, including ethics, philosophy, narrative studies, and cultural analysis, into AI-enabled health technologies. Records were imported into a centralized, web-based screening workspace for duplication and subsequent evaluation. Screening was conducted in two stages (title/abstract followed by full-text review) against predefined eligibility criteria. Titles, abstracts, and full-text articles were screened by a single reviewer using predefined eligibility criteria. Data extraction and thematic coding were conducted by the same reviewer and subsequently reviewed through collaborative discussion among the authors to enhance analytic rigor and ensure alignment with the study objectives. In line with the study’s objective of aggregating pre-validated, high-quality evidence across diverse clinical contexts, inclusion was restricted to scoping and systematic reviews that synthesized digital humanities applications in child and adolescent mental health services. This review was conducted in accordance with the PRISMA Extension for Scoping Reviews (PRISMA-ScR) guidelines [33], checklist see Supplementary Materials. Given the interdisciplinary nature of Digital Humanities and the inherent variability in clinical intervention models and theoretical orientations, a review of secondary research was selected as the most robust approach. This methodology facilitates a comprehensive mapping of the existing knowledge base, allowing for the synthesis of evidence across a diverse landscape of study designs while identifying critical gaps for future inquiry. A review protocol was developed to guide the review process; however, it was not formally registered.

The inclusion and exclusion criteria were established a priori to ensure that selected studies directly addressed the intersection of digital humanities and AI-enabled digital mental health interventions for children and adolescents. Consistent with the objective of synthesizing higher-order evidence, only systematic and scoping reviews were eligible because these designs facilitate structured synthesis across heterogeneous studies and support identification of recurring themes and conceptual frameworks. Eligible reviews were required to examine AI-enabled digital mental health interventions, including machine learning, predictive algorithms, large language models, or AI-based decision-support systems, while incorporating digital humanities perspectives as a central analytical or methodological framework. Humanities-informed perspectives included ethical, cultural, philosophical, or social dimensions that informed the design, implementation, evaluation, or interpretation of AI technologies in mental health contexts.

Studies were included only if they satisfied all predefined eligibility criteria. Publications focusing exclusively on non-AI digital interventions, lacking a substantive digital humanities perspective, addressing general AI ethics without application to digital mental health, or employing non-review publication formats (e.g., editorials, opinion papers, letters, or conference abstracts) were excluded. Full-text articles that did not meet one or more eligibility criteria were excluded (Figure 1). These criteria ensured that the final sample of 17 studies comprised methodologically rigorous review-level evidence suitable for thematic synthesis of the design, governance, implementation, and ethical dimensions of AI-enabled digital mental health technologies.

Data from each included study was systematically extracted using a structured charting framework developed a priori (see Table 1). The data extraction template captured study characteristics, primary healthcare domain, categories of AI-enabled technologies examined, humanities disciplines or theoretical frameworks engaged, and principal findings relevant to integration, outcomes, and governance. An inductive thematic analysis was conducted to synthesize findings across the included sources. Consistent with established review methodology, formal critical appraisal of included studies was not conducted. The objective of this review was to map the breadth, characteristics, and thematic patterns of the existing literature rather than to evaluate the methodological quality or risk of bias of individual studies [33,34,35]. Thematic analysis was selected as a flexible and robust method for identifying recurring patterns, key concepts, and thematic convergence within a heterogeneous body of literature, including empirical studies, theoretical papers, and conceptual frameworks [36]. This approach enabled a nuanced examination of how humanities disciplines inform the design, governance, and implementation of AI-based digital mental health tools, particularly within mental health contexts. The analysis began with descriptive coding of key variables, such as represented humanities disciplines, AI technologies, integration mechanisms, and stakeholder roles, which were then grouped into higher-order themes through iterative comparison and constant engagement with the data [37]. Coding and theme development were carried out collaboratively to enhance analytical rigor and ensure alignment with the review’s objectives. The final synthesis yielded six overarching themes that illustrated the contributions, value propositions, and implementation challenges of integrating the humanities into AI-based health technologies. In this context, thematic analysis served not only to summarize the state of the literature but also to uncover conceptual gaps, unresolved tensions, and emergent opportunities at the intersection of technological innovation and humanistic inquiry.

## 3. Results

The literature search identified 1648 records through PubMed and an additional 74 records through Elicit semantic searches. After duplicate removal, 1672 records underwent title and abstract screening. Of these, 90 full-text articles were assessed for eligibility, and 17 studies met the inclusion criteria for thematic synthesis. The included studies comprised systematic reviews, scoping reviews, and meta-analyses addressing digital mental health interventions, AI ethics, participatory design, implementation, and governance across school, clinical, community, and digital health settings, and synthesized findings from randomized trials, observational studies, qualitative research, and conceptual analyses. Six recurring themes were identified across the evidence base: engagement, participatory co-design, human oversight, equity, ethical governance, and implementation. Evidence concerning intervention effectiveness was strongest in systematic reviews and meta-analyses, whereas findings related to ethics, governance, equity, and implementation were derived primarily from scoping reviews and conceptual syntheses.

The first theme concerned engagement. Across the included reviews, engagement was associated with intervention modality, developmental appropriateness, stigma, and family or social context [24,49]. Several studies also reported that intervention characteristics, including gamification and the availability of human support, influenced engagement and intervention outcomes [7,8].

The second theme involved participatory co-design. Studies described the use of stakeholder advisory boards, community partnerships, and iterative feedback throughout intervention development [1,47]. Liverpool et al. [38] additionally identified co-production as a common component of intervention design and implementation.

The third theme focused on human oversight. Reviews consistently reported that interventions incorporating human support demonstrated improved effectiveness compared with fully automated approaches [8]. Ethical reviews also emphasized maintaining meaningful human control in AI-assisted decision-making to support patient safety, accountability, and professional responsibility [39,50].

The fourth theme addressed equity. Several studies reported inconsistent collection and reporting of sociodemographic information, limiting the assessment of disparities and reducing generalizability [11]. Reviews of universal interventions also reported limited attention to structural inequities and inclusive design [46]. Ethical reviews further identified algorithmic bias as a recurring concern associated with AI implementation [51].

The fifth theme concerned ethical governance. Reviews consistently identified principles such as beneficence, autonomy, justice, transparency, privacy, and accountability when discussing AI implementation [39,40]. Several studies also reported fragmented regulatory frameworks and the absence of harmonized standards for AI governance [50]. Youth-focused reviews further identified privacy, validation, and online safety as recurring considerations [43].

The sixth theme addressed implementation. Across the included studies, implementation commonly involved institutional readiness, workforce training, sustainability, and contextual adaptation [38,41]. Reviews also reported considerable variability in implementation despite evidence supporting remote and digitally delivered interventions [45]. Similar findings were reported in chronic disease populations, where implementation varied across healthcare settings [42].

Across the 17 included reviews, these themes were reported in diverse settings, including schools, outpatient clinics, community programs, and digital platforms. Although evidence supporting symptom reduction was strongest in randomized and meta-analytic studies, recurring findings across the literature also addressed engagement, participatory design, human oversight, equity, ethical governance, and implementation within AI-enabled youth mental health interventions.

## 4. Discussion

This scoping review synthesizes review-level evidence on the integration of digital humanities within AI-enabled youth mental health interventions. The six themes identified in the Results collectively indicate that humanities-informed perspectives are consistently incorporated into the design, governance, and implementation of digital mental health technologies. Rather than representing isolated ethical considerations, these perspectives appear throughout the development of AI-supported interventions.

Across the included reviews, humanities contributions converge around three interrelated domains: design, governance, and implementation. Within the design domain, engagement extends beyond usability to include developmental appropriateness, participatory co-design, and cultural responsiveness [38,47,49]. These observations are consistent with previous evidence demonstrating that meaningful engagement and stakeholder participation improve intervention acceptability and implementation [1,24]. Within governance, the literature consistently emphasizes transparency, accountability, and meaningful human oversight as important considerations for AI-assisted decision making [39,40,50]. Within implementation, institutional readiness, workforce capacity, and contextual adaptation are repeatedly identified as factors influencing successful deployment of digital interventions [38,41,42,45].

The review also highlights differences in how humanities perspectives are positioned within AI development. Some studies describe humanities-informed approaches primarily as practical strategies for improving usability and trust, whereas others frame them as broader perspectives for examining the ethical, cultural, and sociotechnical assumptions embedded within AI systems [39,51]. This distinction suggests that digital humanities contributes not only to intervention development but also to critical reflection on the values and assumptions that shape AI-supported care.

Equity emerged as another consistent finding across the literature. Inconsistent reporting of sociodemographic characteristics limits assessment of disparities and reduces generalizability [11]. Ethical analyses further identify algorithmic bias as reflecting broader structural inequities embedded within data collection and institutional practices [51]. These findings support the need for culturally responsive data practices and more transparent reporting in future AI-enabled mental health research.

Several limitations should be considered. This study was designed as a scoping review to map the breadth of the literature rather than evaluate methodological quality or intervention effectiveness. Consistent with established scoping review methodology, formal critical appraisal was not conducted. Although a review protocol was developed to guide the review process, it was not formally registered. Furthermore, screening, data extraction, and initial coding were conducted by a single reviewer and subsequently reviewed collaboratively by the research team. While this approach enhanced the consistency of thematic interpretation, independent duplicate screening would further strengthen methodological rigor. Finally, the evidence base remains heterogeneous, with relatively limited empirical research examining ethics-of-care frameworks, generative AI, and large language models within youth mental health systems.

Future research should evaluate humanities-informed AI approaches through longitudinal implementation studies, equity-focused analyses, and real-world evaluations of emerging AI technologies. Additional research is also needed to examine how digital humanities can inform governance frameworks, culturally responsive implementation, and recovery-oriented mental health systems across diverse youth populations.

## 5. Conclusions

This scoping review demonstrates that digital humanities provides an important interdisciplinary perspective for understanding the design, governance, and implementation of AI-enabled youth mental health interventions. Across 17 review-level studies, humanities-informed approaches were consistently associated with participatory design, human oversight, ethical governance, equity, and context-sensitive implementation. Although the evidence base remains heterogeneous, the findings suggest that integrating humanities-informed perspectives may strengthen the development of more ethical, equitable, and developmentally responsive AI-supported mental health technologies.

Future research should move beyond conceptual discussions toward empirical evaluation of humanities-informed AI approaches, particularly in relation to implementation, equity, and emerging generative AI technologies. Such work will help determine how digital humanities can contribute to the responsible development and deployment of AI-enabled mental health systems for children and adolescents.

## Figures and Tables

**Table 1:** Overview of the final 17 studies included in the review.

Authors (Year)	Aim	Primary Healthcare Domain	Humanities Disciplines	Key Findings
Bergin et al. [1]	To critically evaluate the design characteristics and reporting quality of preventive digital mental health interventions targeting children and adolescents.	General Child & Adolescent Mental Health	Participatory Design	Digital health interventions (DHIs) have frequently been highlighted as one way to respond to increasing levels of mental health problems in children and young people, but Bergin et al. (2020) identified substantial variability in design and reporting. Limited transparency in development and engagement strategies constrains reproducibility and scalability [1]. Strengthening user-centered, developmentally appropriate design and standardized reporting is essential to advance preventive digital mental health interventions.
Liverpool et al. [38]	To synthesize evidence on the factors that influence engagement with digital mental health interventions among children and adolescents.	General Child & Adolescent Mental Health	Narrative Medicine/Digital Storytelling	Engagement is a central yet fragile determinant of the effectiveness of digital mental health interventions among youth, shaped by personalization, usability, human support, and developmental relevance, but hindered by usability issues, a lack of co-design, and contextual misalignment.
Murphy et al. [39]	To provide a comprehensive overview of the ethical considerations, frameworks, and debates surrounding the application of AI in health and public health contexts.	Diagnostics, precision medicine	Ethics	A scoping review of 103 studies (including 22 grey literature) revealed ethical concerns related to AI use in health care: privacy & security, trust in AI applications, accountability & responsibility for the use of AI technology, and adverse consequences of bias in algorithms and the data used to train them. Largely missing from the literature was the ethics of AI in global health, especially in low- and middle-income countries.
Möllmann et al. [40]	To provide a comprehensive overview of the ethical challenges, concerns, and frameworks associated with implementing AI technologies in digital health contexts.	General healthcare, geriatrics	Ethics	50 articles were reviewed by 5 major ethical principles. 3 principles received the most research: beneficence (48%), non-maleficence (70%), autonomy (30%), justice (48%), explicability (34%). The review highlights potential areas with little empirical evidence and calls for further research to address these gaps.
Sakellari et al. [41]	To explore and synthesize the role of digital health promotion approaches in supporting mental health and wellbeing in primary school populations.	General Child & Adolescent Mental Health	Implicit Humanities Alignment	Mental health challenges among primary school children continue to represent a significant concern. Although the current evidence base is limited, available studies suggest that web-based interventions can improve teachers’ knowledge and attitudes while supporting positive behavioral outcomes in children. The scarcity of robust evidence underscores the need for further research in this area.
Sasseville et al. [42]	To provide a timely and comprehensive overview of how digital health interventions are used to support mental health management in populations with chronic physical health conditions.	Youth Mental Health	Narrative Medicine/Digital Storytelling	Digital health interventions show promise in improving mental health outcomes in chronic disease populations, particularly for depression and anxiety. However, substantial heterogeneity, limited long-term evidence, and implementation challenges, including engagement and integration into care, constrain conclusions. Further rigorous and standardized research is needed to support scalability and sustained effectiveness.
Wies et al. [43]	To provide a comprehensive scoping review of the ethical opportunities and risks related to the use of digital mental health tools in children and adolescent populations.	General Child & Adolescent Mental Health	Bioethics/Ethics	Digital mental health interventions for young people offer significant promise in improving access and early support, but raise critical ethical challenges related to privacy, safety, consent, equity, and the preservation of therapeutic relationships.
Yu et al. [44]	To develop an evidence and gap map (EGM) that provides a structured overview of the types, distribution, and strength of evidence for Mental Health and Psychosocial Support interventions targeting children and adolescents in lower-middle-income countries	Youth Mental Health	Implicit Humanities Alignment	The evidence base for child and adolescent mental health interventions in LMICs is uneven and concentrated in specific contexts and modalities, with substantial gaps in early childhood, long-term outcomes, equity, and system-level implementation research.
Fischer-Grote et al. [45]	To synthesize and quantitatively assess (via meta-analysis) the impact of digitally delivered mental health interventions on psychological outcomes in young populations during the post-pandemic period.	Youth Mental Health	Implicit Humanities Alignment	Online and remote mental health interventions for youth demonstrate small-to-moderate effectiveness, particularly when guided by human support, highlighting both their scalability and the importance of structured, supported implementation models.
Kirvin-Quamme et al. [11]	To map and critically evaluate current practices in the collection and reporting of sociodemographic characteristics within studies of digital mental health interventions.	General Child & Adolescent Mental Health	Implicit Humanities Alignment	Sociodemographic data reporting in digital mental health research is inconsistent and insufficiently standardized, limiting the ability to assess equity and raising concerns about bias and generalizability in digital and AI-enabled interventions.
Di Pierdomenico et al. [46]	To provide a comprehensive overview of universally delivered (population-level) digital mental health interventions targeting children and adolescents, focusing on their characteristics, implementation, and outcomes.	Youth Mental Health	Implicit Humanities Alignment	Universal digital mental health interventions for youth show modest preventive benefits but are constrained by engagement challenges, limited co-design, insufficient attention to equity, and a lack of long-term outcome evidence.
Kaur et al. [47]	To design, refine, and assess a community-partnered, school-based digital intervention that addresses emotion regulation difficulties among autistic children.	General Child & Adolescent Mental Health	Participatory Design	Co-developed digital mental health interventions for children on the autism spectrum are feasible and acceptable in school settings, with effectiveness contingent on stakeholder involvement, contextual adaptation, and guided implementation.
Liverpool et al. [38]	To conduct a systematic overview of reviews that consolidates and updates the evidence base on the effectiveness, design, and implementation of digital mental health interventions for youth.	Youth Mental Health	Narrative Medicine/Digital Storytelling	Digital mental health interventions for children and young people demonstrate modest effectiveness, with outcomes strengthened by human support and co-production, but are constrained by engagement challenges, variable evidence quality, and unresolved implementation and safety issues.
Takizawa et al. [8]	To conduct a meta-analysis assessing whether universally delivered digital interventions (i.e., non–risk-targeted, population-level approaches) improve mental health outcomes in children and adolescents.	Youth Mental Health	Implicit Humanities Alignment	Universal digital mental health interventions for children and adolescents produce small but significant preventive effects, with outcomes moderated by intervention design, engagement features, and the presence of human support.
Walder et al. [8]	To conduct a systematic review and meta-analysis of randomized controlled trials (RCTs) assessing whether digitally delivered interventions can reduce symptoms of social anxiety and support prevention efforts in young populations.	Youth Mental Health (Anxiety/Depression)	Implicit Humanities Alignment	Digital mental health interventions, particularly CBT-based and guided formats, are effective in reducing social anxiety symptoms among youth and young adults, with outcomes influenced by engagement, support, and intervention structure.
Babbitt et al. [48]	To describe the planned methodology for a systematic review evaluating the diagnostic accuracy, study quality, and applicability of digital question-and-answer–based mental health assessment tools across age groups. This publication is a study protocol.	General Child & Adolescent Mental Health	Implicit Humanities Alignment	Not applicable.
Chew et al. [49]	To provide a comprehensive synthesis of the types, functions, and effectiveness of digital mental health interventions designed for children and family systems.	Youth Mental Health	Narrative Medicine/Digital Storytelling	Digital mental health interventions for children and families improve access and show modest effectiveness, but are limited by fragmented child–parent approaches, engagement challenges, and insufficient attention to contextual and equity considerations.

Note. The table summarizes key characteristics of the included studies, including study aim or purpose, primary healthcare domain, humanities disciplines, and key findings.

## Data Availability

No new data were created or analyzed in this study. Data sharing is not applicable to this article.

## Supplementary Materials

The following supporting information can be downloaded at: https://www.mdpi.com/article/10.3390/children13070967/s1, PRISMA-ScR Checklist.
