# Supplementary material for: Digital Humanities in Child and Adolescent Mental Health Services: A Review
Source: Children (Basel). 2026 Jul 22;13(7):967. doi: 10.3390/children13070967 (PMC13406150; doi:10.3390/children13070967)
Supplement: Supplementary file 1 [file children-13-00967-s001.zip › PRISMA-ScR Checklist.pdf]

PRISMA-ScR Checklist

Section	Item	Checklist Item	Reported	Location in Manuscript
Title	1	Identify the report as a scoping review.	Yes	Title
Abstract	2	Provide a structured summary including background, objectives, eligibility criteria, sources of evidence, charting methods, results, and conclusions.	Yes	Abstract
Introduction: Rationale	3	Describe the rationale for the review in the context of what is already known.	Yes	Introduction
Introduction: Objectives	4	Provide an explicit statement of the questions and objectives being addressed.	Yes	Introduction
Methods: Protocol & registration	5	Indicate whether a review protocol exists, and if and where it can be accessed.	No	Methods & Data Extraction and Synthesis
Methods: Eligibility criteria	6	Specify eligibility criteria.	Yes	Eligibility Criteria
Methods: Information sources	7	Describe all information sources in the search.	Yes	Search Strategy
Methods: Search	8	Present a full electronic search strategy for at least one database.	Partial	Search Strategy
Methods: Selection of sources of evidence	9	Describe the process for selecting sources of evidence.	Yes	Eligibility Criteria
Methods: Data charting process	10	Describe the methods of charting data from included sources.	Yes	Data Extraction and Synthesis
Methods: Data items	11	List and define all variables for which data were sought.	Yes	Data Extraction and Synthesis
Methods: Critical appraisal of individual sources of evidence	12	If done, provide a rationale for the critical appraisal of sources.	Not performed	Data Extraction and Synthesis
Synthesis of results	13	Describe the methods of handling and summarizing the data that were charted.	Not performed	Data Extraction and Synthesis

Results: Selection of sources of evidence	14	Give numbers of sources screened, assessed for eligibility, and included, with reasons for exclusions.	Yes	Figure 1
Results: Characteristics of sources of evidence	15	Give characteristics of included sources of evidence.	Yes	Results and Table 1
Results: Critical appraisal within sources of evidence	16	Present relevant data from included sources of evidence.	Yes	Results and Table 1
Results: individual sources of evidence	17	For each included source of evidence, present the relevant data that were charted that relate to the review questions and objectives.	Yes	Results & Thematic Domains
Results: Synthesis of the findings	18	Summarize and/or present the charting results as they relate to the review questions and objectives.	Yes	Results & Thematic Domains
Discussion: Summary of evidence	19	Summarize the main results including an overview of concepts and themes.	Yes	Discussion
Discussion: Limitation	20	Discuss limitations of the scoping review process.	Yes	Limitations and Future Research
Discussion: Conclusion	21	Provide general interpretation and implications of the results.	Yes	Conclusion
Funding	22	Describe sources of funding for the included sources and the review itself.	Yes	Author Note
